# Portuguese version of the fear of COVID-19 scale – psychometric study

**DOI:** 10.1192/j.eurpsy.2021.696

**Published:** 2021-08-13

**Authors:** C. Cabaços, A.T. Pereira, P. Paredes, S. Almeida, A. Araujo, R. Sousa, A. Macedo

**Affiliations:** 1 Psychiatry Department, Centro Hospitalar e Universitário de Coimbra, Coimbra, Portugal; 2 Institute Of Psychological Medicine, Faculty Of Medicine, University of Coimbra, coimbra, Portugal; 3 Faculty Of Medicine, University of Coimbra, Coimbra, Portugal; 4 Usf Coimbra Centro, USF Coimbra Centro, Coimbra, Portugal

**Keywords:** COVID-19, Fear of COVID-19

## Abstract

**Introduction:**

More than in other conditions, fear is associated with infectious diseases, and is directly associated with its transmission rate, morbidity and mortality. High levels of fear can affect the individual’s ability to think clearly, react proportionately and make rational decisions in the context of COVID-19. Recently, Mertens et al. (2020) developed the Fear of Covid-19 Scale (FCV-19S) to measure this construct.

**Objectives:**

To analyse the psychometric properties of the FCV-19S Portuguese version, namely construct validity, internal consistency and convergent validity.

**Methods:**

A community sample of 234 adults (75.6% women; mean age= 29.53±12.51; range:16-71) completed an on-line survey with the Portuguese versions of the FCV-19S, the Covid-19 Perceived Risk Scale (CPRS) and the Depression Anxiety Stress Scale (DASS-21).The total sample was randomly divided in two sub-samples: sample A (n=117) was used to perform an exploratory factor analysis/EFA; sample B (n=117) to make a confirmatory factor analysis/CFA.

**Results:**

EFA resulted in one component. CFA revealed that the unifactorial model presented acceptable fit indexes (X2/df=3.291; CFI=.977; GFI=.932; TLI=.919; p[RMSEA≤.01]=.091). Cronbach alpha was α=.855. The total score significantly correlated with Covid-19 Perceived Risk (r=.529, p<.01) and with anxiety from DASS-21 (r=.132, p<.05).

**Conclusions:**

This study provides preliminary evidence for the validity and reliability of the Portuguese version of FCV-19S, which will be used in an ongoing research project on the relationship between fear of Covid-19, personality, cognitive processes and adherence to public health measures to contain the pandemic.

